# Protective Psychic Factors for Aging and Their Association with Dementia Risk Factor in a sample of Argentinian Older Adults

**DOI:** 10.1002/alz70858_104995

**Published:** 2025-12-24

**Authors:** Maria Celeste López Moreno, Leticia Vivas, Renata Diaz Colodrero, Estela Benitez, Manuel Obando Ulloa, Guillermina Quenan, Melania Lope, Carla Perez, María Martha Richard´s, Daiana Bario

**Affiliations:** ^1^ Institute of Basic and Applied Psychology and Technology (IPSIBAT), National University of Mar del Plata (UNMDP), Psychology Faculty, Mar del Plata, Buenos Aires, Argentina; ^2^ Faculty of Health Sciences and Social Work of the National University of Mar del Plata (UNMDP), Mar del Plata, Buenos Aires, Argentina; ^3^ National Council of Scientific and Technical Research (CONICET), Mar del Plata, Buenos Aires, Argentina

## Abstract

**Background:**

Population aging is a growing global phenomenon. In Mar del Plata, Argentina, 21% of the population is over 60 years old (2022 Census). An Argentinian study has indicated that the percentage of dementia cases that would be potentially preventable with appropriate management of the risk factor is 59.6% (Calandri et al., 2023). However, the impact of subjective variables as personality traits, emotions, resilience, and beliefs about aging and its relationship with risk factors remains unknown. It is necessary to consider these psychic factors when analyzing protective factors for dementia. This study analyzes protective psychological factors in older adults and their association with dementia risk factors.

**Method:**

The study included 66 older adults (90.9% women), participating in the University Program for Older Adults (PUAM) and community centers of Mar del Plata. The average age was 69.4 years, with a medium‐high educational level. The FAPPREN (Protective Psychic Factors for Aging) questionnaire (Zarebski et al., 2017) was administered, along with individual interviews collecting data about educational level, comorbidities, physical activity, socialization, and alcohol and tobacco consumption. The Kendall correlation model was applied to examine the associations between the FAPPREN score and dementia risk factors.

**Result:**

With a maximum FAPPREN score of 30, the sample mean score was 26.3. Kendall's Tau correlation analysis showed that higher psychic protective factors were associated with fewer comorbidities (*p* < .001), higher educational levels (*p* < .05), and a tendency of positive correlation with frequency of socialization (*p* = .06). No significant association was identified with physical activity (*p* = .90), tobacco consumption (*p* = .19) and alcohol consumption (*p* = .31).

**Conclusion:**

These results provide evidence in favor of the comprehensive approach to dementia prevention. Intervening on protective psychological factors, such as flexibility, self‐care and adaptation, promotes overall health. Additionally, educational level emerged as a protective factor. Addressing dementia risk factors from a holistic perspective is essential, fostering psychological resources to cope with change, combating ageism, and promoting positive perceptions of aging throughout the life course.

